# Supplementary material for: Maternal BMI During Lactation Is Associated with Major Protein Compositions in Early Mature Milk
Source: Nutrients. 2024 Nov 7;16(22):3811. doi: 10.3390/nu16223811 (PMC11597192; doi:10.3390/nu16223811)
Supplement: Supplementary file 1 [file nutrients-16-03811-s001.zip › nutrients-3272353-supplementary.pdf]

Supplementary Materials

Table S1. Other characteristics of mothers and infants

Variables	Descriptive value
**Education status (%)**	
Senior high and below	18 (25.7%)
Junior high and above	52 (74.3%)
**Per capita monthly household income (%)**	
<5000 Yuan	18 (25.7%)
≥5000 Yuan	52 (74.3%)
**Pregnancy times (%)**	
1	38 (54.3%)
≥2	32 (45.7%)
**Delivery times (%)**	
1	49 (70.0%)
≥2	21 (30.0%)
**Gestational age (weeks, M(P25, P75), %)**	39 (38, 40)
Premature infant	0
Term infant	70 (100.0%)
**Infant growth retardation (%)**	1 (1.4%)
**Infant low body weight (%)**	0

Growth retardation and low body weight in infants were evaluated according to height-for-age (HAZ) and weight for age (WAZ), respectively.HAZ < -2.0 was defined as growth retardation and WAZ < -2.0 was defined as low body weight.

Table S2. TP level according to characteristics

Variables	Lg TP	*P*
Overall	0.097±0.088	
**Area**		0.731
West	0.100±0.081	
South	0.118±0.110	
North	0.092±0.051	
East	0.085±0.101	
**Delivery mode**		0.926
Cesarean	0.096±0.084	
Eutocia	0.098±0.091	
**GWG**		0.284
Inadequate	0.062±0.091	
Appropriate	0.105±0.088	
Excessive	0.106±0.086	
**Infant gender**		0.757
Male	0.094±0.096	
Female	0.101±0.079	
**Gestational hypertension**		0.071
Yes	0.175±0.114	
No	0.093±0.085	
**Gestational diabetes**		0.214
Yes	0.137±0.121	
No	0.093±0.084	

P:Student t-test or ANOVA test.

Table S3. Level of each whey protein and proportion to TP according to characteristics ($\bar{x} \pm s$)

**Variables**	**Lg α-La**	***P***	**α-La/TP(%)**	***P***	**Lg LF**	***P***	**LF/TP(%)**	***P***	**Lg OPN**	***P***	**OPN/TP(%)**	***P***
Overall	2.564±0.123		29.7±4.9		1.939±0.156		7.3±2.1		1.287±0.122		1.6±0.3	
**Area**		0.851		0.079		**0.033**		**0.002**		0.585		0.080
West	2.579±0.129		30.6±5.0		1.970±0.132		7.7±2.2		1.256±0.104		1.5±0.3	
South	2.541±0.124		26.8±4.4		1.842±0.186		5.6±1.9		1.289±0.125		1.5±0.3	
North	2.565±0.087		30.0±4.1		1.931±0.109		7.1±1.5		1.294±0.124		1.6±0.3	
East	2.566±0.144		30.7±5.3		1.985±0.159		8.2±1.9		1.310±0.134		1.7±0.3	
**Delivery mode**		0.546		0.396		0.576		0.738		0.962		0.853
Cesarean	2.552±0.124		29.0±5.2		1.953±0.134		7.4±1.7		1.288±0.123		1.6±0.3	
Eutocia	2.571±0.124		30.1±4.8		1.931±0.168		7.2±2.3		1.287±0.122		1.6±0.3	
**GWG**		0.152		0.390		0.442		0.909		0.802		0.556
Inadequate	2.505±0.122		28.2±5.4		1.889±0.151		7.1±2.2		1.270±0.111		1.7±0.3	
Appropriate	2.582±0.124		30.4±5.0		1.945±0.137		7.3±2.2		1.296±0.118		1.6±0.3	
Excessive	2.571±0.118		29.5±4.5		1.956±0.182		7.4±2.1		1.284±0.135		1.5±0.3	
**Infant gender**		0.694		0.311		0.274		0.233		0.571		0.757
Male	2.569±0.127		30.2±4.9		1.920±0.158		7.0±2.0		1.280±0.132		1.6±0.4	
Female	2.557±0.120		29.0±5.0		1.962±0.153		7.6±2.2		1.297±0.109		1.6±0.3	
**Gestational hypertension**		**0.021**		0.090		0.438		0.595		0.495		0.412
Yes	2.700±0.168		33.8±4.4		1.998±0.129		6.7±1.0		1.328±0.133		1.5±0.3	
No	2.556±0.117		29.5±4.9		1.935±0.157		7.3±2.2		1.285±0.121		1.6±0.3	
**Gestational diabetes**		**0.030**		**0.025**		0.890		0.210		0.744		0.410
Yes	2.659±0.111		33.6±5.3		1.931±0.137		6.3±1.1		1.302±0.128		1.5±0.3	
No	2.553±0.121		29.3±4.7		1.940±0.159		7.4±2.2		1.286±0.122		1.6±0.3	

P:Student t-test or ANOVA test.

Table S4. Level of each casein protein and proportion to TP according to characteristics ($\bar{x} \pm s$)

Variables	Lg $α_{S1}$-CN	*P*	$α_{S1}$-CN/TP(%)	*P*	Lg β-CN	*P*	β-CN/TP(%)	*P*	Lg κ-CN	*P*	κ-CN/TP(%)	*P*
Overall	1.719±0.113		4.2±0.6		2.670±0.122		37.9±6.0		1.601±0.125		3.3±0.7	
**Area**		0.379		**<0.001**		0.742		**0.024**		**0.013**		**<0.001**
West	1.743±0.101		4.4±0.6		2.685±0.102		38.8±5.1		1.651±0.129		3.6±0.7	
South	1.676±0.119		3.7±0.5		2.642±0.125		33.8±5.7		1.519±0.079		2.6±0.5	
North	1.722±0.080		4.3±0.5		2.664±0.15		38.7±8.4		1.588±0.106		3.2±0.7	
East	1.725±0.137		4.4±0.5		2.680±0.120		39.5±3.6		1.624±0.137		3.5±0.6	
**Delivery mode**		0.447		0.134		0.189		**0.025**		0.752		0.688
Cesarean	1.733±0.105		4.4±0.6		2.696±0.099		40.1±5.0		1.608±0.121		3.3±0.7	
Eutocia	1.711±0.118		4.1±0.6		2.655±0.132		36.7±6.3		1.598±0.129		3.3±0.8	
**GWG**		**0.016**		**0.014**		0.093		0.198		0.544		0.953
Inadequate	1.640±0.093		3.8±0.6		2.605±0.107		35.3±5.6		1.567±0.107		3.3±0.8	
Appropriate	1.743±0.104		4.4±0.6		2.691±0.108		38.9±5.0		1.605±0.114		3.3±0.7	
Excessive	1.728±0.121		4.2±0.6		2.675±0.139		38.1±7.3		1.614±0.149		3.3±0.8	
**Infant gender**		0.700		0.820		0.397		0.454		0.151		0.108
Male	1.714±0.123		4.2±0.6		2.659±0.138		37.4±6.8		1.582±0.124		3.2±0.7	
Female	1.725±0.101		4.2±0.6		2.684±0.099		38.5±4.8		1.626±0.125		3.4±0.8	
**Gestational hypertension**		**0.021**		0.098		**0.023**		0.105		**0.024**		0.244
Yes	1.845±0.160		4.7±0.7		2.803±0.125		42.7±4.4		1.737±0.123		3.7±0.7	
No	1.711±0.107		4.2±0.6		2.662±0.118		37.6±6.0		1.593±0.121		3.3±0.7	
**Gestational diabetes**		0.142		0.262		0.120		0.246		0.963		0.230
Yes	1.779±0.109		4.5±0.9		2.738±0.105		40.4±7.0		1.599±0.127		3.0±0.6	
No	1.712±0.113		4.2±0.6		2.662±0.122		37.7±5.9		1.602±0.126		3.3±0.8	

P:Student t-test or ANOVA test.

Table S5. level of **each** MFGM protein according to characteristics ($\bar{x} \pm s$)

**Variables**	**Lg BTN**	***P***	**Lg PAS6/7**	***P***	**Lg FABP**	***P***	**Lg XOR**	***P***
Overall	0.791±0.170		0.294±0.149		-0.099±0.239		1.092±0.165	
**Area**		0.297		0.436		**0.034**		0.629
West	0.842±0.187		0.291±0.141		-0.094±0.247		1.092±0.181	
South	0.786±0.151		0.302±0.145		0.049±0.231		1.139±0.155	
North	0.729±0.159		0.244±0.157		-0.174±0.180		1.071±0.162	
East	0.794±0.171		0.327±0.154		-0.155±0.240		1.072±0.162	
**Delivery mode**		0.833		0.885		0.879		0.858
Cesarean	0.785±0.195		0.298±0.158		-0.104±0.236		1.087±0.180	
Eutocia	0.794±0.157		0.292±0.146		-0.095±0.243		1.094±0.158	
**GWG**		0.305		0.337		0.090		0.240
Inadequate	0.797±0.142		0.313±0.148		-0.033±0.240		1.100±0.124	
Appropriate	0.760±0.170		0.266±0.136		-0.165±0.241		1.058±0.172	
Excessive	0.830±0.181		0.322±0.166		-0.043±0.221		1.133±0.169	
**Infant gender**		0.894		0.191		0.784		0.984
Male	0.789±0.184		0.273±0.139		-0.106±0.238		1.092±0.170	
Female	0.794±0.155		0.320±0.159		-0.090±0.244		1.091±0.160	
**Gestational hypertension**		0.908		0.194		0.879		0.832
Yes	0.801±0.055		0.389±0.061		-0.116±0.141		1.109±0.033	
No	0.790±0.175		0.288±0.151		-0.098±0.244		1.090±0.169	
**Gestational diabetes**		0.941		0.965		0.709		0.886
Yes	0.796±0.181		0.296±0.16		-0.066±0.318		1.083±0.175	
No	0.791±0.17		0.294±0.149		-0.102±0.231		1.092±0.165	

P:Student t-test or ANOVA test.

Table S6. Variance inflation factor of variables in models

Variables	VIF_1	Df_1	VIF_2	Df_2
**Maternal BMI**	1.187	1	1.155	3
**Covariates**				
Lactation stage (days)	1.150	1	1.152	1
Maternal age (years)	1.223	1	1.265	1
Area	1.078	3	1.078	3
Delivery mode	1.149	1	1.161	1
GWG	1.149	2	1.224	2
Infant gender	1.146	1	1.091	1
Gestational hypertension	1.163	1	1.203	1
Gestational diabetes	1.217	1	1.225	1

VIF_1:continuous change; VIF_2:category change.

Table S7. Results of model diagnostics

Models	R^2	F	*P*	*D-W*
**Lg TP**				
Continuous change	0.393	3.069	0.002	1.531
Category change	0.424	2.896	0.002	1.701
**Lg α-La**				
Continuous change	0.447	3.840	<0.001	1.783
Category change	0.478	3.603	<0.001	1.845
**α-La/TP (%)**				
Continuous change	0.320	2.231	0.022	2.228
Category change	0.340	2.024	0.033	2.168
**Lg LF**				
Continuous change	0.312	2.158	0.027	1.661
Category change	0.347	2.085	0.027	1.721
**LF/TP (%)**				
Continuous change	0.342	2.466	0.011	2.036
Category change	0.372	2.330	0.013	1.975
**Lg OPN**				
Continuous change	0.253	1.606	**0.116**	1.812
Category change	0.327	1.911	0.045	1.892
**OPN/TP (%)**				
Continuous change	0.203	1.207	**0.301**	2.181
Category change	0.234	1.198	**0.303**	2.120
**Lg $α_{S1}$-CN**				
Continuous change	0.455	3.958	<0.001	1.501
Category change	0.484	3.688	<0.001	1.610
**$α_{S1}$-CN/TP (%)**				
Continuous change	0.436	3.673	<0.001	2.134
Category change	0.478	3.597	<0.001	2.067

Continued Table S7. Results of model diagnostics

Models	R^2	F	*P*	*D-W*
**Lg β-CN**				
Continuous change	0.315	2.183	0.025	1.779
Category change	0.373	2.338	0.013	1.973
**β-CN/TP (%)**				
Continuous change	0.297	2.010	0.040	2.253
Category change	0.334	1.969	0.038	2.345
**Lg κ-CN**				
Continuous change	0.358	2.646	0.007	1.895
Category change	0.378	2.391	0.011	1.853
**κ-CN/TP (%)**				
Continuous change	0.388	3.009	0.003	2.271
Category change	0.414	2.769	0.004	2.219
**Lg BTN**				
Continuous change	0.155	0.872	**0.579**	2.364
Category change	0.254	1.340	**0.215**	2.406
**Lg PAS6/7**				
Continuous change	0.242	1.516	**0.145**	2.366
Category change	0.290	1.603	**0.108**	2.434
**Lg FABP**				
Continuous change	0.274	1.794	**0.071**	2.198
Category change	0.313	1.788	**0.064**	2.263
**Lg XOR**				
Continuous change	0.151	0.843	**0.607**	2.443
Category change	0.235	1.209	**0.296**	2.518

P: F-test.

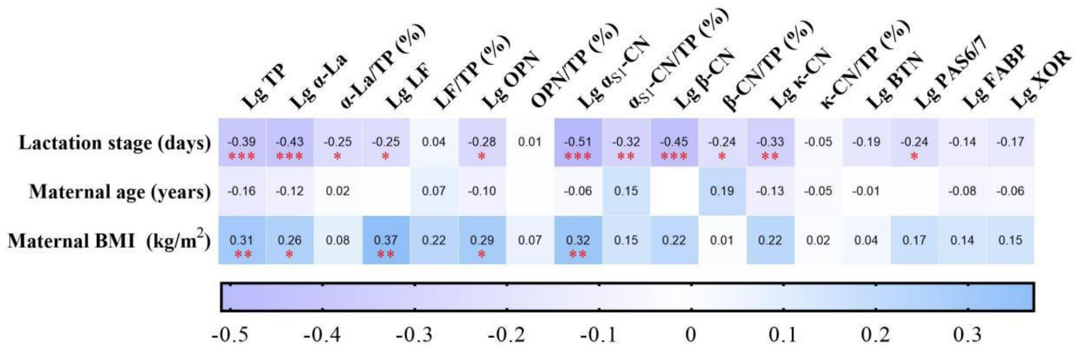

Figure S1. Correlation of common logarithm levels and proportions of each protein in the HM and other continuous variables

Lactation stage: Spearman correlation; Maternal age: Pearson correlation.

** $p < 0.05$, ** $p < 0.01$, ***$p < 0.001$.*

Maternal BMI	Lg BTN	β×10⁻³ [95%CI]	P	Lg PAS6/7	β×10⁻³ [95%CI]	P	Lg FABP	β×10⁻³ [95%CI]	P	Lg XOR	β×10⁻³ [95%CI]	P
**Continuous change**		0.4[−14.1,15.0]	0.954		7.6[−4.5,19.6]	0.213		12.9[−6.0,31.8]	0.177		5.5[−8.6,19.6]	0.436
**Comparison between groups**												
Normal:Underweight		4.9[−174.0,183.8]	0.956		92.9[−60.1,245.9]	0.229		11.1[−229.9,252.1]	0.927		35.5[−139.7,210.7]	0.686
Overweight:Underweight		−71.1[−256.0,113.7]	0.444		110.0[−48.1,268.1]	0.169		38.3[−210.7,287.3]	0.759		−17.6[−198.6,163.3]	0.846
Obesity:Underweight		120.0[−97.0,337.0]	0.273		**203.6[18.1,389.1]**	**0.032**		220.1[−72.1,512.4]	0.137		165.5[−46.9,377.9]	0.124
Overweight:Normal		−76.1[−176.0,23.9]	0.133		17.1[−68.4,102.5]	0.691		27.2[−107.4,161.8]	0.687		−53.1[−151.0,44.7]	0.281
Obesity:Normal		115.0[−26.3,256.4]	0.109		110.7[−10.2,231.5]	0.072		**209.0[18.7,399.4]**	**0.032**		130.0[−8.4,268.4]	0.065
Obesity:Overweight		**191.1[46.0,336.2]**	**0.011**		93.6[−30.5,217.7]	0.136		181.8[−13.6,377.3]	0.068		**183.1[41.1,325.2]**	**0.012**
	-200 -100 0 100 200 300			0 100 200 300			-200 0 200 400			-200 -100 0 100 200 300		

Figure S2. Associations between maternal BMI and MFGM proteins' level according to multiple linear regression analyses. The model was adjusted by lactation stage (days), maternal age (years), residential area (west, south, north, east), delivery modes (cesarean, eutocia), GWG(inadequate,appropriate,excessive), infant gender (male, female) and conditions of gestational complications (hypertension, diabetes).
